# The Feasibility of Patient-Specific Circulating Tumor DNA Monitoring throughout Multi-Modality Therapy for Locally Advanced Esophageal and Rectal Cancer: A Potential Biomarker for Early Detection of Subclinical Disease

**DOI:** 10.3390/diagnostics11010073

**Published:** 2021-01-05

**Authors:** Christopher Boniface, Christopher Deig, Carol Halsey, Taylor Kelley, Michael B. Heskett, Charles R. Thomas, Paul T. Spellman, Nima Nabavizadeh

**Affiliations:** 1 Cancer Early Detection Advanced Research (CEDAR) Center, Division of Oncological Sciences, Knight Cancer Institute, Oregon Health & Science University (OHSU), 2720 SW Moody Ave., Portland, OR 97201, USA; boniface@ohsu.edu (C.B.); spellmap@ohsu.edu (P.T.S.); 2 Department of Biomedical Engineering, Oregon Health & Science University (OHSU) School of Medicine, 3181 SW Sam Jackson Park Rd, Portland, OR 97239, USA; 3 Department of Radiation Medicine, Oregon Health & Science University (OHSU), 3181 SW Sam Jackson Park Rd, KPV4, Portland, OR 97239, USA; deig@ohsu.edu (C.D.); thomasch@ohsu.edu (C.R.T.J.); 4 Department of Molecular and Medical Genetics, Oregon Health & Science University (OHSU) School of Medicine, 3181 SW Sam Jackson Park Rd, Portland, OR 97239, USA; halseyc@ohsu.edu (C.H.); kelleyt@ohsu.edu (T.K.); heskett@ohsu.edu (M.B.H.)

**Keywords:** liquid biopsy, ctDNA, cell free DNA, non-operative management, neoadjuvant therapy

## Abstract

As non-operative management (NOM) of esophageal and rectal cancer is becoming more prevalent, blood-biomarkers such as circulating tumor DNA (ctDNA) may provide clinical information in addition to endoscopy and imaging to aid in treatment decisions following chemotherapy and radiation therapy. In this feasibility study, we prospectively collected plasma samples from locally advanced esophageal (*n* = 3) and rectal cancer (*n* = 2) patients undergoing multimodal neoadjuvant therapy to assess the feasibility of serial ctDNA monitoring throughout neoadjuvant therapy. Using the Dual-Index Degenerate Adaptor-Sequencing (DIDA-Seq) error-correction method, we serially interrogated plasma cell-free DNA at 28–41 tumor-specific genomic loci throughout therapy and in surveillance with an average limit of detection of 0.016% mutant allele frequency. In both rectal cancer patients, ctDNA levels were persistently elevated following total neoadjuvant therapy with eventual detection of clinical recurrence prior to salvage surgery. Among the esophageal cancer patients, ctDNA levels closely correlated with tumor burden throughout and following neoadjuvant therapy, which was associated with a pathologic complete response in one patient. In this feasibility study, patient- and tumor-specific ctDNA levels correlated with clinical outcomes throughout multi-modality therapy suggesting that serial monitoring of patient ctDNA has the potential to serve as a highly sensitive and specific biomarker to risk-stratify esophageal and rectal cancer patients eligible for NOM. Further prospective investigation is warranted.

## 1. Introduction

As non-operative management (NOM) of locally-advanced esophageal and rectal cancer following chemotherapy and radiation therapy is more widely adopted [1,2], a sensitive and specific biomarker of sub-clinical tumor burden has the potential of further assisting in the assessment of patients best suited for an upfront non-operative approach or for detecting early sub-clinical recurrences in patients in need of salvage surgery in the surveillance period [3]. Circulating tumor DNA (ctDNA) has been extensively investigated for its diagnostic and prognostic utility as such a biomarker [4,5,6,7,8], however, the longitudinal application of ctDNA monitoring throughout multi-modality therapy (systemic therapy, radiotherapy, and/or surgery) has been less extensively studied [4]. Undetectable ctDNA following definitive treatment has been shown to be associated with pathologic complete response (pCR) and improved outcomes, particularly in the neoadjuvant setting for breast cancer [9,10]; however, the feasibility of serial ctDNA measurements in the neoadjuvant setting for rectal and esophageal cancer has not been previously reported.

**Figure 1:**
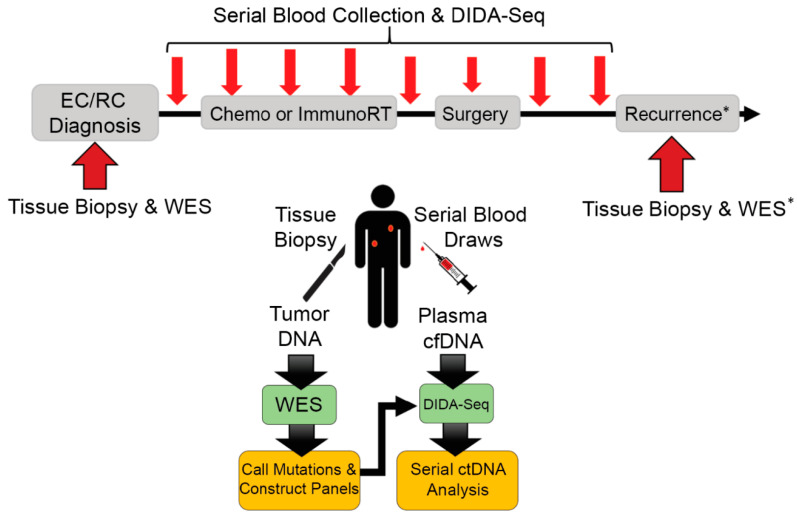
Patient treatment and sample collection schema for blood draws and solid tissue biopsies. Solid tissue biopsies were collected after initial diagnosis of esophageal or rectal cancer (ER/RC) and prior to treatment for whole-exome sequencing (WES). Blood was collected prior to Table S1. month intervals during treatment and surgery, and ~3-month intervals during follow-up monitoring. Mutations were called between solid tissue biopsy WES and matched buffy coat WES and used to construct patient-specific sequencing library enrichment panels. Cell-free DNA (cfDNA) isolated from blood draws was sequenced using Dual-Index Degenerate Adaptor-Sequencing (DIDA-Seq) at sites identified in each patient’s tumor biopsy to retrospectively determine circulating-tumor DNA (ctDNA) prevalence. * Patient 3 had a solid tissue biopsy of a metastasis which was also analyzed by WES and variants were included in their patient-specific panel.

Our ctDNA monitoring technique, called DIDA-Seq (Dual-Indexed, Degenerate Adaptor-Sequencing), combines unique-molecular indexing (UMI)-based error correction with custom hybridization capture at many genomic loci of somatic variants previously identified by whole-exome sequencing of the patient’s tumor tissue. This method allows the detection of ctDNA in the blood with very high accuracy (one error in 10k–50k observations) and sensitivity (0.005–0.02% minimum variant allele frequency [11]). This study aims to test the feasibility of using patient and tumor-specific ctDNA monitoring to assess residual disease in five esophageal and rectal cancer patients during and after multimodal neoadjuvant therapy (Figure 1).

## 2. Materials and Methods

### 2.1. Patient Enrollment, Tissue Processing, and DNA Extraction

In this feasibility study, human specimens and data (including blood, tumor tissue, and clinical information) were prospectively acquired from participants with locally-advanced esophageal (*n* = 3) or rectal cancer (*n* = 2) undergoing definitive multimodal therapy after their informed written consent (Oregon Health & Science University Institutional Review Board Study #10163, first approved 19 October 2017). Plasma samples were collected at baseline, throughout therapy and surveillance. Biopsy tissue was collected at diagnosis and recurrence (Figure 1). Blood draws were serially collected and fractionated for cfDNA isolation using the “double spin” method (≤40 mL, a range of 6–40 mL, in 5 × 6 mL or 4 × 10 mL, purple-capped EDTA tubes) [8,11]. DNA was extracted from FFPE, plasma, and buffy coat using commercially available kits (see below). Within 6 h of collection, blood plasma was isolated by first spinning whole blood at 1000 g for 10 min, separating the top plasma layer into 1-mL aliquots, then spinning those aliquots at 15,000 g for 10 min, transferring the supernatant to cryovials, and storing at −80 °C. Fixed formalin paraffin-embedded biopsies and tumor-tissue were collected, and DNA extraction was carried out using QIAgen FFPE DNA extraction kit (QIAGEN, Redwood City, CA, USA). DNA was extracted from plasma and buffy coat using Macherey-Nagel NucleoSnap (Macherey-Nagel GmbH & Co., Duren, Germany) and QIAgen Blood and Tissue kits, respectively. All DNA extractions were quantified using the Qubit 3 fluorometric quantification system (ThermoFisher Scientific, Waltham, MA, USA) and size distribution was checked with a BioAnalyzer 2100 (Agilent Technologies, Santa Clara, CA, USA). DNA isolated from FFPE samples and buffy coat were fragmented by sonication to 150 bp using a Covaris E220 (Covaris Inc., Woburn, MA, USA) prior to library preparation (cfDNA was not fragmented prior to ligation).

### 2.2. Whole-Exome Sequencing Library Preparation

Whole-exome sequencing (WES) libraries were prepared from tissue biopsies using 100–500 ng of sonicated FFPE or buffy coat DNA and the KAPA Hyper-Prep Kit (KAPA Biosystems, Capetown, South Africa) with the Agilent SureSelect XT Target Enrichment System and Human All Exon V5 capture baits (Agilent Technologies, Santa Clara, CA, USA). Next generation sequencing was carried out using the Illumina NextSeq 500 platform by our institution’s Massively Parallel Sequencing Shared Resource to an average, de-duplicated depth of 329X and 121X for tumor and buffycoat matched-normal libraries, respectively (Supplementary Table S3).

### 2.3. Somatic Mutation Calling and Design of Tumor-Specific Capture Panels

FastQ data files were aligned and processed using BWA MEM (0.7.12, http://bio-bwa.sourceforge.net/). Somatic variants were called using aligned BAM files and MuTect (1.1.4, GATK, Broad Institute, Cambridge, MA, USA) between tumor and the patient’s matched normal from blood buffy coat [12]. All WES BAM files can be found in the Sequence Read Archive (www.ncbi.nlm.nih.gov/sra) under the BioProject accession number PRJNA637431 [13]. Single nucleotide variant (SNV) calls were filtered out if they were present in the dbSNP database (www.ncbi.nlm.nih.gov/projects/SNP). SNVs were filtered by frequency (requiring >1% variant allele frequency and >3 supporting reads in the tumor, and <2% variant allele frequency in the matched normal) and depth (requiring ≥30X coverage in the tumor and ≥14X coverage in the matched normal) and were further assessed and hand-curated using Oncotator [14] and IGV [15] software. For tumor-specific capture targets, approximately 50 SNVs were chosen for each patient based on inferred clonality, sequence context, and potential functional impact. To address concerns over properly representing cell subpopulations, intronic mutations were included in each panel. Tumor-specific hybrid capture panels were constructed by querying the human reference genome (GRCh37/hg19) for the 120 bp surrounding the target loci of interest. The resulting nucleotide sequences were submitted to IDT DNA (Coralville, IA, USA) to generate biotinylated bait oligos using the NGS Discovery Pools tool. Mutation sites and bait oligo sequences are described in Supplementary Table S1.

### 2.4. DIDA-Seq Library Preparation and Sequencing

DIDA-Seq error-correction libraries were prepared similarly to what is previously described and sequenced on Illumina platforms. Briefly, 30–100 ng of cell-free DNA was input into the Kapa Biosystems Hyper Prep kit with custom DIDA-Seq adaptors followed by hybridization capture using the IDT xGen Hybridization and Wash Kit using a single, 18 h capture incubation step instead of the double-incubation steps previously described [8,11]. Libraries were sequenced on either the Illumina HiSeq 2500, paired-end 100 bp, with dual 14-bp indexing cycles or the Illumina NextSeq 500, paired-end 70 bp with dual 14-bp indexing cycles. All DIDA-Seq BAM files can be found in the Sequence Read Archive (www.ncbi.nlm.nih.gov/sra) under the BioProject accession number PRJNA637431 [13].

### 2.5. Evaluation of Tumor-Specific Capture Panel Performance and CtDNA Prevalence

The error-correction pipeline for analyzing DIDA-Seq data was based on the duplex sequencing pipeline with substantial modification to be compatible with our data [16]. The DIDA-Seq computational pipeline was implemented as previously described [11] and the variant allele frequency (VAF) was determined for each mutation at each time point by dividing the number of mutant error-corrected (i.e., consensus) reads by the total number of consensus reads at that site and multiplying by 100 (note that all VAFs are reported as a percentages in Supplementary Tables). The aggregate VAFs for each time point were calculated by summing the mutant consensus reads at all sites interrogated, dividing that by the total number of consensus reads across all sites and multiplying by 100. Each hybrid capture panel was evaluated using unrelated patient cfDNA samples as negative controls. We sequenced each patient time-point library to a mean, consensus read depth of 5.2kX (range = 159X to 23.4kX) per site-of-interest. We sequenced each negative control library to an average per-site consensus read depth of 43.6kX coverage (range = 3.9kX to 127kX) with an average per-site error rate of 0.0067% or 1 error in 15k site-of-interest observations (range = 1 error in 2.7k to 125k site-of-interest observations) providing an average limit of detection of 0.016% VAF (i.e., the mean of the lowest statistically significant VAF from Patients 2–5, see Supplementary Table S4). When we aggregated negative control site-of-interest consensus read counts for each panel, we calculated an average per-panel error rate of 0.0057%, or 1 error in 17.7k observations with a range of 1 in 12.5k to 22.9k based on the assumption that mutant consensus reads found in the negative control were caused by PCR or sequencer error (see Supplementary Table S2 for panel-specific error rates). We compared the mutation-specific VAF in the patient’s plasma at each time-point to the VAF of the same site in the set of pooled negative controls using the Weitzman overlapping coefficient [17] (see “Significance Tests for CtDNA Measurements” below). A *p*-value was generated for each site, as well as all sites aggregated by tumor-specific panel, using the overlap coefficient between the beta distributions of the sample and the negative control read counts as described below. Any individual site with greater than 0.05% VAF in the negative controls was omitted from evaluation of ctDNA levels in the respective target patient. Data points having a *p*-value of 0.05 or less were considered significantly different from the negative controls, effectively determining our lower limit of detection given the total sequencing depth at each time point. To correct for differences in cell-free DNA concentration between blood draws, the aggregate VAF was converted into human genome equivalents per mL (hGE/mL) of plasma by the following equation:

$$\frac{cfDNA\;concentration\left(\frac{ng}{ml\;plasma}\right)}{0.003\left(\frac{ng}{genome}\right)}*variant\;allele\;frequency\;=Mutant\;genomes\;per\;mL\;plasma\;\left(or\;hGE/mL\;plasma\right)$$

### 2.6. Significance Tests for CtDNA Measurements

The significance of ctDNA measurements (i.e., mutant consensus reads) at each time point, as compared to the panel’s negative control, was determined prior to conversion to human genome equivalents per ml (hGE/mL) plasma and is dependent on the sequencing depth at each site at that time point. A Bayesian approach was used to test the null-hypothesis that the sample VAF and negative control VAF were generated from the same distribution. This statistical approach was used because we assumed that a higher sample size (i.e., deeper sequencing) confers a more accurate parameter estimate (i.e., 100 mutant reads in 100,000 is more accurate than 1 in 100). Therefore, a Beta distribution was created for the sample and for the negative control (Equations (1) and (2)), setting the “a” and “b” parameter values to the number of variant reads and number of reference reads, respectively. Next, the Weitzman overlapping coefficient [17] (Equation (3)) was used to measure the similarity between the sample and negative control distributions to create a significance value. In cases where the number of mutant consensus reads was greater than zero but the estimated *p*-value was also greater than 0.05, we determined the minimum number of mutant consensus reads (given the number of total consensus reads), for which the cumulative binomial distribution is greater than or equal to the error rate of the given sites as determined by the negative control (error-rate = mutant consensus reads in negative control/total consensus reads in the negative control). If the observed number of mutant consensus reads exceeded this value, we considered it to be marginally significant and therefore above the limit of detection (see Supplementary Table S4 for *p*-values and binomial test results for each panel at each time point). Note that the Weitzman overlapping coefficient method can result in low *p*-values (<0.05) if the sample VAF ≈ negative control VAF but the depth of the negative control is much greater (>100-fold) than the depth of the sample. In such cases, the ctDNA measurement was considered below the limit of detection if the VAF of the sample was equal to or less than that of the negative control (e.g., Supplementary Table S4, Patient 2, time point #1).

(1) $$X_{sample}\;\sim \;Beta\left(a_{sample},b_{sample}\right)$$

(2) $$X_{neg\_ctrl}\;\sim \;Beta\left(a_{neg\_ctrl},b_{neg\_ctrl}\right)$$

(3) $$\int min\left[\;f_{neg\_ctrl}\left(x\right),\;f_{sample}\left(x\right)\right]dx$$

## 3. Results

### 3.1. Elevated ctDNA Levels Are Associated with Recurrence in Rectal Adenocarcinoma with Clinically-Useful Lead Time

**Figure 2:**
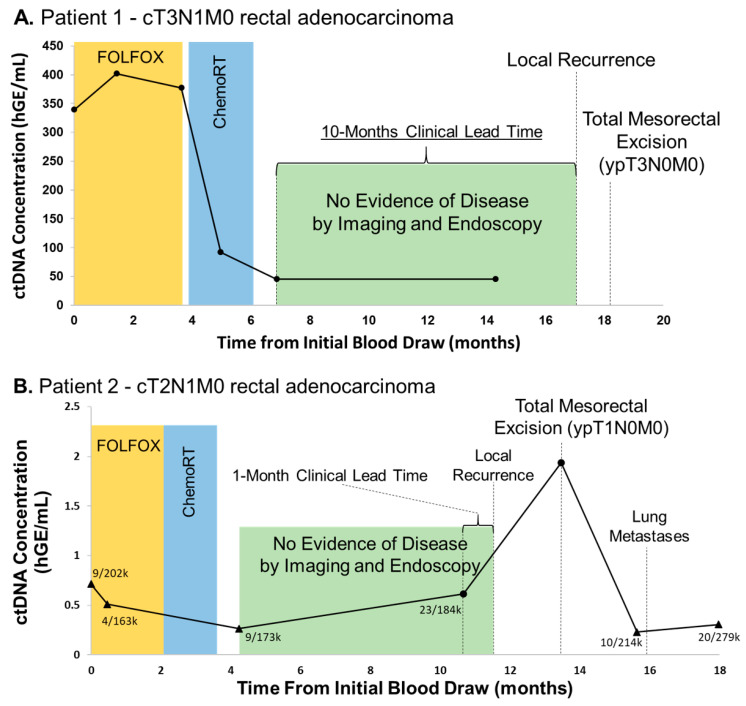
Rectal cancer patients with detectable post-treatment ctDNA eventually had local recurrence. Serial ctDNA levels were retrospectively analyzed using DIDA-Seq and using patient-specific capture panels. Aggregate variant allele frequency (VAF) was converted to human genome equivalents per ml (hGE/mL) of plasma and plotted over treatment course. (**A**) A 28-site capture panel was used for Patient 1 and (**B**) a 35-site capture panel was used for Patient 2. Statistical significance, as compared to a negative control, was determined at each time point. CtDNA values not significantly different from negative controls are indicated (triangle) and aggregate mutant reads/total reads are reported. Statistical significance was determined prior to converting aggregate VAF to hGE/mL plasma.

Patient 1 is a 33 year old female who presented with cT3N1M0 distal rectal adenocarcinoma and enrolled on an unrelated phase II trial evaluating the efficacy of total neoadjuvant therapy (eight cycles of FOLFOX chemotherapy and long-course chemoradiation) followed by non-operative management for clinical complete responders based on MRI and endoscopy (NCT02008656) [13]. Whole exome sequencing (WES) of a pretreatment tissue biopsy revealed 81 non-synonymous single nucleotide variants (SNVs, Supplementary Table S3). DIDA-Seq of 28 loci was used to monitor ctDNA levels throughout the patient’s treatment course. CtDNA levels decreased fivefold during four months of total neoadjuvant therapy (Figure 2A). She was without clinically detectable disease for six months following total neoadjuvant therapy and proceeded with NOM per the trial; however, ctDNA levels remained elevated. Eleven months following total neoadjuvant therapy, endoscopic surveillance revealed a biopsy-confirmed recurrence and the patient underwent salvage total mesorectal excision (TME).

Patient 2 is a 59 year old male who presented with cT2N1M0 mid-rectal adenocarcinoma and enrolled on the aforementioned phase II study. WES of this patient’s tumor biopsy found 106 total non-synonymous SNVs and 35 sites were used to assess ctDNA levels in blood draws. At baseline and following total neoadjuvant therapy, ctDNA levels were not considered significantly above negative control, however mutant reads were present (Figure 2B). Similarly, this patient also proceeded with NOM given clinical complete response seen on endoscopy and imaging. However, ctDNA levels were detectable eight months following the completion of total neoadjuvant therapy one month prior to biopsy-proven local recurrence, and peaked at the time of salvage TME. Following TME, ctDNA levels again returned to below the limits of detection in spite of later oligometastatic progression. Unfortunately, the performance of this patient’s capture panel in the negative control was the lowest of all five panels which resulted in decreased overall sensitivity at the time of oligometastatic progression (see Supplementary Table S2, “Aggregate error rate (%)”).

### 3.2. CtDNA Levels Are Associated with Tumor Burden and Progression in Oligometastatic Esophageal Cancer

**Figure 3:**
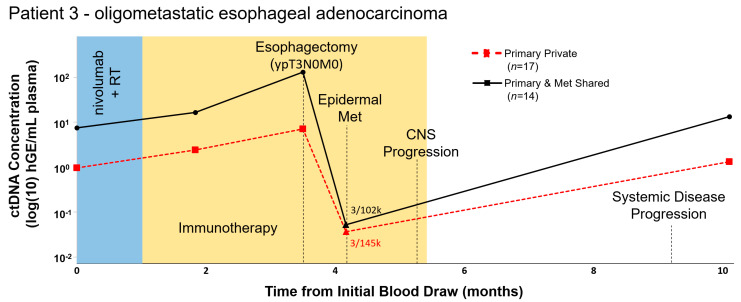
Oligometastatic esophageal adenocarcinoma cancer patient with primary-only oligoprogression had elevated ctDNA levels associated with systemic disease progression. In Patient 3, whole-exome sequencing of both the primary tissue biopsy and a subsequent metastatic dermal lesion revealed a high mutation burden and 45% overlap in mutation profiles. Serial ctDNA levels were retrospectively analyzed using DIDA-Seq and using patient-specific capture panels. Aggregate variant allele frequency (VAF) was converted to human genome equivalents per ml (hGE/mL) of plasma and plotted in log10-scale over treatment course. Plot shows ctDNA monitoring using mutations either private to the primary tissue biopsy (*n* = 17, solid black line) or shared between the primary tissue biopsy and the biopsy of the metastatic dermal lesion (*n* = 14, dashed red line). Statistical significance, as compared to a negative control, was determined at each time point. CtDNA values not significantly different from negative controls are indicated (triangle) and aggregate mutant reads/total reads are reported. Statistical significance was determined prior to converting aggregate VAF to hGE/mL plasma.

Patient 3 is a 72 year old male with oligometastatic esophageal cancer who presented with metastatic disease 2 years prior and had received extensive therapy under an immunotherapy trial. Given oligoprogression at the primary site only (distal esophagus), tumor board recommendations were for the patient to undergo neoadjuvant therapy prior to esophagectomy, at which time he was enrolled on our feasibility study. WES revealed significant intertumoral heterogeneity with only 45% of mutations shared and panel sites were selected to represent both shared and private mutations. Using DIDA-Seq, we assessed 17 mutations found only in the primary tissue biopsy and 14 mutations shared between that tumor and a subsequent metastasis (Figure 3). Increasing ctDNA levels throughout neoadjuvant therapy were consistent with clinical non-response. CtDNA levels became undetectable post-esophagectomy but were again elevated seven months following surgery, concordant with clinical progression.

### 3.3. Undetectable ctDNA Is Associated with Pathologic Complete Response (pCR) Following Tri-Modality Therapy for Esophageal Adenocarcinoma

**Figure 4:**
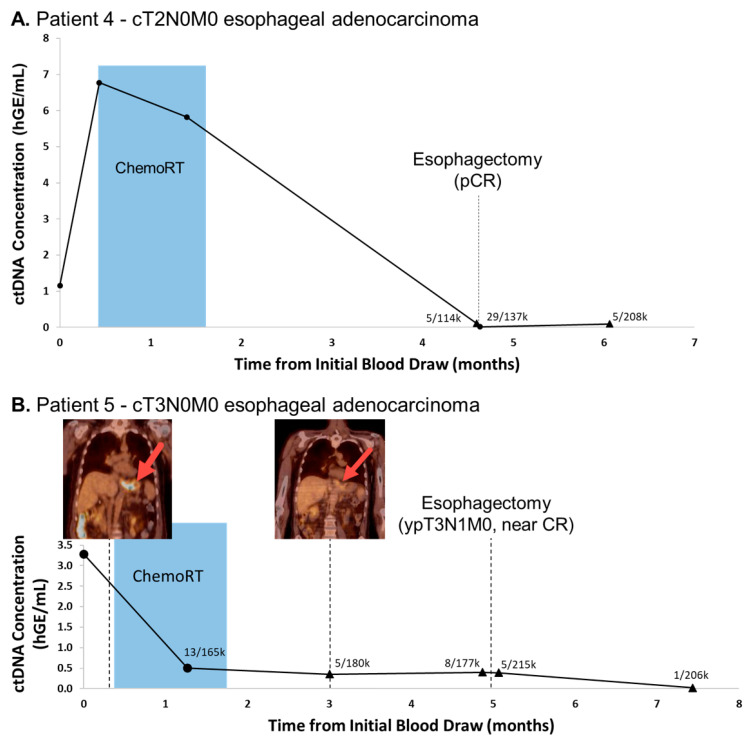
Esophageal adenocarcinoma cancer patients had significant declines in ctDNA during and following neoadjuvant chemoradiation. Patient 4 (**A**) had surgical confirmation of pathologic complete response (pCR) and Patient 5 (**B**) had near complete response (CR). Serial ctDNA levels were retrospectively analyzed using DIDA-Seq and using patient-specific capture panels. Aggregate variant allele frequency (VAF) was converted to human genome equivalents per ml (hGE/mL) of plasma and plotted over treatment course. A 39-site capture panel was used for Patient 4 and a 41-site capture panel was used for Patient 5. 18F-FDG-PET/CT showed reduced tumor size and avidity (red arrows) corresponding to near complete response in Patient 5 (B, inset). Statistical significance, as compared to a negative control, was determined at each time point. CtDNA values not significantly different from negative controls are indicated (triangle) and aggregate mutant reads/total reads are reported. Statistical significance was determined prior to converting aggregate VAF to hGE/mL plasma.

Patient 4 is a 61 year old male with a history of cT2N0M0 distal esophageal adenocarcinoma who underwent neoadjuvant chemoradiation and esophagectomy. WES revealed 585 non-synonymous mutations and 39 sites were interrogated in blood draws by our capture panel. CtDNA levels declined during neoadjuvant therapy, associated with reduced tumor size and avidity on PET-CT, and were near the limit of detection (i.e., indeterminate as compared negative control values, see Methods) with 5 mutant reads in 114k total reads immediately prior to surgery, and 29 mutant reads in 137k total reads immediately following surgery (Figure 4A). Surgical pathology confirmed a pCR and ctDNA levels remained undetectable as compared to the negative control at final follow-up 6 weeks following his esophagectomy.

Patient 5 is a 69 year old male with cT3N0M0 distal esophageal adenocarcinoma who received neoadjuvant chemoradiation prior to esophagectomy with surgical pathology confirming a near-complete response. WES found 135 non-synonymous mutations and 41 genomic site were included in the ctDNA panel. As with Patient 4, ctDNA levels in this patient were elevated prior to treatment, but quickly fell below the limit of detection during chemoradiation with concurrent reduced tumor size and avidity on PET-CT. CtDNA levels remained statistically insignificant at final follow-up 10 weeks following esophagectomy with no clinical evidence of disease at that time (Figure 4B). Eight months later, the patient was found to have a malignant pleural effusion; however plasma was unable to be collected to evaluate the recurrence of ctDNA.

## 4. Discussion

Here, we have demonstrated the feasibility of using patient- and tumor-specific ctDNA monitoring throughout neoadjuvant therapy and surveillance, identifying that such an assay may have the potential to detect sub-clinical disease and more precisely select candidates for organ preservation or those who may benefit from early salvage resection. Given the morbidity and mortality of large oncologic surgeries, notably esophagectomy, non-operative management for complete responders to neoadjuvant therapy is intriguing and is an active area of investigation [1,18]. Current standard of care for locally-advanced esophageal or rectal cancer consists of neoadjuvant therapy followed by planned surgical resection irrespective of response or biomarker readout. Up to 50% of esophageal squamous cell carcinoma patients exhibit a pCR following neoadjuvant chemoradiation. This has been consistently shown to predict for better disease-free survival and overall survival [19,20,21,22,23,24] with a meta-analysis identifying a 33–36% overall survival benefit when a pCR is achieved [25]. Given the morbidity and mortality associated with esophagectomy [26,27,28], avoidance of resection is desirable in those who are at low risk for having residual disease. Furthermore, there is growing evidence in the rectal cancer literature that regimented clinical assessment of patients following neoadjuvant chemoradiation can potentially identify those who are clinical complete responders, allowing avoidance of immediate surgery [29,30,31]. A multicenter U.S. trial recently presented preliminary findings testing this hypothesis and found that a watch and wait strategy in a large proportion of patients achieving pCR after neoadjuvant therapy resulted in organ preservation without compromising survival [18].

Many providers are reluctant to adopt this approach broadly given the poor sensitivity and specificity of clinical response assessments. Current post-neoadjuvant clinical assessment for both esophageal and rectal cancers consists only of direct endoscopic visualization and anatomic/functional imaging (CT, PET/CT, and MRI). These tests have difficulty differentiating small regions of treatment-related inflammation or fibrosis from persistent tumor and vice-versa. Multiple studies have examined the concordance rates between these tests and pathology specimens, none of which have exhibited sufficient sensitivity or specificity to accurately identify true complete responders. In rectal cancer, functional MRI has shown great promise with a substantial improvement in sensitivity and specificity (~85% for both) [32]. However, in esophageal cancer assessment of complete response is considerably poor where a combination of endoscopic ultrasound and PET/CT yields only a specificity of 30% [33]. Moreover, as lymph node metastases are still identified in up to 8% of patients with pCR of the primary tumor [34], a more robust and unambiguous biomarker for assessment of complete clinical response is needed and will drastically impact treatment decision making.

There are limited published data on ctDNA quantification during and after neoadjuvant therapy and its correlation with treatment response and suitability for surgery [4]. CtDNA has been shown useful in the detection of minimal residual disease following breast conservation therapy for women with early-stage breast cancer, with detection of ctDNA in plasma after completion of curative therapy predicting metastatic relapse with high accuracy [35]. In a similar study for Stage II and III rectal cancer patients receiving tri-modality therapy with planned surgery, the presence of tumor-specific ctDNA during post-neoadjuvant chemoradiation was highly predictive for disease recurrence despite adjustment for stage, CEA levels, and use of adjuvant therapy [36]. Additionally, in a heterogeneous cohort of esophageal cancer patients receiving chemoradiation either in the neoadjuvant or definitive setting, post-chemoradiation panel-based mutation detection of ctDNA was associated with tumor progression, metastasis, and shorter survival [37]. The results and feasibility of our patient- and tumor-specific ctDNA assay in this cohort of patients adds to this body of literature and the impact of ctDNA as a useful response assessment biomarker.

There are some limitations to our ctDNA methodology, however. The DIDA-Seq method we have utilized achieves high sensitivity by sequencing select sites to great depth with UMI-based error correction. Consequently, three limiting factors must be considered: (1) hypermutated source tissue, (2) tumor heterogeneity, and (3) variability in performance between selected loci. In Patient 3, mutations shared between the primary and subsequent metastasis were 20-fold more prevalent than those private to the primary and therefore easier to detect. However, a clinical application of our assay for monitoring ctDNA would typically be limited to the mutations found only in the initial tissue biopsy. This highlights the importance of designing patient panels that are representative of both treatment-responsive and treatment-resistant cancer cell populations. Furthermore, poor site selection may contribute to high, panel-specific error-rates as seen in Patient 2, which had the worst performing panel of all five patients (Supplementary Table S2). For example, it is possible that mutant reads found in this patient at time points prior to surgery, which were determined to be below the panel’s limit of detection, were indeed true positives and thus would have provided additional clinical lead time. As sequencing costs decrease, it may be feasible to routinely monitor cell-free DNA for every mutation identified by exome- or whole-genome sequencing of tumor biopsies, potentially mitigating such issues.

In this feasibility study, patient- and tumor-specific ctDNA analysis throughout multi-modality therapy for esophageal and rectal cancer patients was shown to be feasible and potentially useful in the assessment of treatment response which would have particular utility in watch and wait and organ preservation strategies. Further investigation with a larger and more homogenous cohort is warranted.

## Data Availability

The data used for analysis in this report consisted of de-identified patient clinical information and next-generation sequencing data using both custom capture and whole-exome capture library preparations. Patient clinical data are presented in the report and sequencing data (BAM files) are available on the Sequence Read Archive website (www.ncbi.nlm.nih.gov/sra) under the BioProject accession number PRJNA637431 (https://www.ncbi.nlm.nih.gov/sra/PRJNA637431).

## Supplementary Materials

The following are available online at https://www.mdpi.com/2075-4418/11/1/73/s1, Supplementary Table S1: Patient-specific hybrid capture targets and bait oligo sequences; Supplementary Table S2: Patient-specific hybrid capture panel evaluation in negative controls; Supplementary Table S3: WES statistics and mutation counts; Supplementary Table S4: DIDA-Seq cfDNA read counts and statistical evaluation.
